# Pre-treatment with *Bifidobacterium breve* UCC2003 modulates *Citrobacter rodentium-*induced colonic inflammation and organ specificity

**DOI:** 10.1099/mic.0.060830-0

**Published:** 2012-11

**Authors:** James W. Collins, Ali R. Akin, Artemis Kosta, Ning Zhang, Mark Tangney, Kevin P. Francis, Gad Frankel

**Affiliations:** 1 Centre for Molecular Bacteriology and Infection, Division of Cell and Molecular Biology, Flowers Building, Imperial College London, London SW7 2AZ, UK; 2 Caliper – a PerkinElmer Company, Alameda, CA 94501, USA; 3 Cork Cancer Research Centre, BioSciences Institute, University College Cork, Cork, Ireland

## Abstract

*Citrobacter rodentium,* which colonizes the gut mucosa via formation of attaching and effacing (A/E) lesions, causes transmissible colonic hyperplasia. The aim of this study was to evaluate whether prophylactic treatment with *Bifidobacterium breve* UCC2003 can improve the outcome of *C. rodentium* infection. Six-week-old albino C57BL/6 mice were pre-treated for 3 days with *B. breve*, challenged with bioluminescent *C. rodentium* and administered *B. breve* or PBS-C for 8 days post-infection; control mice were either administered *B. breve* and mock-infected with PBS, or mock-treated with PBS-C and mock-infected with PBS. *C. rodentium* colonization was monitored by bacterial enumeration from faeces and by a combination of both 2D bioluminescence imaging (BLI) and composite 3D diffuse light imaging tomography with µCT imaging (DLIT-µCT). At day 8 post-infection, colons were removed and assessed for crypt hyperplasia, histology by light microscopy, bacterial colonization by immunofluorescence, and A/E lesion formation by electron microscopy. Prophylactic administration of *B. breve* did not prevent *C. rodentium* colonization or A/E lesion formation. However, this treatment did alter *C. rodentium* distribution within the large intestine and significantly reduced colonic crypt hyperplasia at the peak of bacterial infection. These results show that *B. breve* could not competitively exclude *C. rodentium*, but reduced pathogen-induced colonic inflammation.

## Introduction

Enterohaemorrhagic *Escherichia coli* (EHEC) is an extracellular zoonotic intestinal pathogen that produces Shiga toxin (Stx), and was responsible for over 1034 human infections in England and Wales in 2009 (Health Protection Agency, 2011). Aside from causing acute gastrointestinal infections, EHEC can cause severe clinical disease syndromes such as haemorrhagic colitis and haemolytic uraemic syndrome (HUS) in humans (Tarr *et al.*, 2005). Treatment of EHEC infections with antibiotics such as trimethoprim/sulfamethoxazole and gentamicin has been demonstrated to increase Stx production and may increase the incidence of HUS (Dundas *et al.*, 2005; McGannon *et al.*, 2010; Panos *et al.*, 2006). However, to date, no suitable therapy or intervention strategy exists for EHEC infections.

Probiotics have been demonstrated as an intervention strategy to prevent the colonization of humans and mice with pathogenic enteric micro-organisms in a process termed competitive exclusion (CE) (Bernet-Camard *et al.*, 1997; Chen *et al.*, 2005; Corr *et al.*, 2007; Fanning *et al.*, 2012; Preidis *et al.*, 2011; Wu *et al.*, 2008). CE is a process in which commensal bacterial species are used to outcompete invading pathogenic micro-organisms through a variety of mechanisms including the production of antimicrobial compounds (Bernet-Camard *et al.*, 1997; Corr *et al.*, 2007), competition for receptor sites on the gastrointestinal mucosa (Chen *et al.*, 2007; Servin & Coconnier, 2003), competition for nutrients (Fuller, 1992) and interference with quorum-sensing signals (Medellin-Peña *et al.*, 2007).

Probiotics are defined as live micro-organisms which, when administered in an adequate amount, provide a health benefit to the host (FAO, 2001). Historically, probiotics are from the genera *Lactobacillus* and *Bifidobacterium* (Felis & Dellaglio, 2007). Bifidobacteria are Gram-positive, obligate anaerobes that colonize the large intestines of humans and mice, and are among the most widely used probiotic bacteria (Picard *et al.*, 2005). To date, significant understanding has been gained regarding the use of probiotics to prevent gastrointestinal infections (Huebner & Surawicz, 2006; Servin & Coconnier, 2003; Servin, 2004). However, the underlying mechanisms in the majority of these studies remain to be elucidated.

The mouse pathogen *Citrobacter rodentium* utilizes a type III secretion system (T3SS) to colonize the human intestinal mucosa via the formation of attaching and effacing (A/E) lesions, and is used as a small animal model of human infection with EHEC (Mundy *et al.*, 2005). *C. rodentium* infection induces transmissible colitis and colonic epithelial cell hyperplasia, and results in a self-limiting disease in C57BL/6 mice which induces sterilizing immunity, preventing reinfection (Mundy *et al.*, 2005).

The *C. rodentium* infection model has been widely adopted to study how probiotics can be used to treat gastrointestinal infections (Chen *et al.*, 2005, 2009; D’Arienzo *et al.*, 2006; Fanning *et al.*, 2012; Gareau *et al.*, 2010; Johnson-Henry *et al.*, 2005; Jones & Knight, 2012; Rodrigues *et al.*, 2012; Wu *et al.*, 2008). Recently, Fanning *et al.* (2012) demonstrated that *Bifidobacterium breve* reduces *C. rodentium* colonization in a BALB/c infection model, and that this protective effect is dependent on the production of an extracellular polysaccharide. In addition, the probiotic yeast *Saccharomyces boulardii* has been demonstrated to reduce *C. rodentium* colonization by modulating T3SS expression (Wu *et al.*, 2008). In contrast, pre-treatment of neonatal and adult mice with individual probiotic strains resuspended in PBS has been shown to reduce the intestinal inflammation associated with *C. rodentium* infection in a manner independent from reduced pathogen colonization (Chen *et al.*, 2009; Gareau *et al.*, 2010; Johnson-Henry *et al.*, 2005; Wu *et al.*, 2008).

Bioluminescence imaging (BLI) is widely used in infectious disease research to monitor the colonization of mice with pathogenic bacteria (Contag *et al.*, 1995; Doyle *et al.*, 2004; Hardy *et al.*, 2004; Wiles *et al.*, 2004) and to assess intervention strategies for bacterial infections including probiotics (Corr *et al.*, 2007; Fanning *et al.*, 2012). Recombinant micro-organisms expressing the bacterial luciferase operon *luxCDABE* from *Photorhabdus luminescens* can be detected non-invasively and monitored longitudinally during an infection through light production (Hardy *et al.*, 2004; Wiles *et al.*, 2004, 2005). Importantly, as bioluminescence (BL) is an energy-dependent process, only live, metabolically active micro-organisms are detected (Szittner & Meighen, 1990). However, standard BLI is limited because it is not possible to determine the exact location of the BL foci *in vivo*; instead, localization of the BL signal is inferred from the surface of the animal where the signal is emitted, or through *ex vivo* analysis of the infected organs (Contag *et al.*, 1995). In contrast, 3D BLI, known as diffuse light imaging tomography (DLIT), is performed by collecting BL images taken using different optical filters in the range of 500–620 nm for imaging of bacterial luciferase. The spectrally filtered BL is then used to reconstruct the BL source, location and intensity, resulting in a quantitative 3D reconstruction of the BL signal (Kuo *et al.*, 2007). These BLI data can then be co-registered with a µCT scan of the entire mouse to give a detailed anatomical localization of the BL source in a technique known as DLIT-µCT.

In this study we used DLIT-µCT and BLI, combined with light and electron microscopy, to determine whether the prophylactic treatment of mice with *B. breve* UCC2003 could impact on *C. rodentium* infection.

## Methods

### 

#### Bacterial strains and media.

The bioluminescent *C. rodentium* derivative ICC180 (Wiles *et al.*, 2004) was grown at 37 °C in Luria–Bertani (LB) medium supplemented with kanamycin (50 µg ml^−1^).

*B. breve* UCC2003 (Cronin *et al.*, 2012) was grown statically at 37 °C under anaerobic conditions (BBL GasPak EZ system) in MRS medium supplemented with 0.05 % cysteine HCL (MRS-C).

#### Mice.

Pathogen-free female 18–20 g, 6–8-week-old albino C57Bl/6 mice were purchased from Charles River. All mice were housed in individually filtered cages with sterile bedding and with sterilized food and water *ad libitum*. All animal experiments were performed in accordance with the Caliper IACUC Ethical Review Committee. Two independent infection experiments were performed with six mice per group.

#### Daily treatment of mice with *B. breve* or PBS.

Mice were inoculated for 3 days prior to *C. rodentium* infection and for a further 8 days post-infection (p.i.) by oral gavage with 200 µl of overnight *B. breve* UCC2003, which was resuspended in PBS supplemented with 0.05 % cysteine HCL (PBS-C) at a cell density of approximately 2×10^9^ c.f.u. (Cronin *et al.*, 2010). Uninfected mice were gavaged with either *B. breve* UCC2003 or PBS-C as controls.

### Oral infection of mice

#### 

##### *C. rodentium* challenge.

At 2 PM on day 10 post initiation of *B. breve* UCC2003 treatment, mice were inoculated by oral gavage with 200 µl of overnight LB-cultured *C. rodentium*, which was resuspended in PBS prior to infection at a cell density of approximately 5×10^9^ c.f.u., and uninfected mice were gavaged with PBS as a control. The numbers of viable bacteria in the inoculum were determined by serial dilution in PBS and spotting in triplicate onto LB agar supplemented with kanamycin (50 µg ml^−1^). Colonization was monitored by the collection of faeces from mice at day 7 p.i. and the numbers of viable bacteria per gram of faeces were enumerated. At day 8 p.i., the mice were euthanized by cervical dislocation, and colonic tissues were collected for microscopic analysis as outlined below.

#### Collection of samples, sample fixation and histopathology.

Segments of terminal colon from each mouse were collected post-mortem from one experiment at day 8 p.i. Tissues were subsequently rinsed of their contents and fixed in 10 % buffered formalin for microscopic examination. Additional colonic segments were fixed in 2.5 % glutaraldehyde for further electron microscopy analysis.

#### Measurement of crypt hyperplasia.

Formalin-fixed tissues were then processed, paraffin-embedded, sectioned at 5 µm, and stained with haematoxylin and eosin (H&E) using standard techniques. H&E-stained tissues were evaluated for crypt hyperplasia microscopically without knowledge of the treatment condition used in the study, and the length of at least 20 well-oriented crypts from each section from all of the mice per treatment group (*n* = 6) was evaluated. H&E-stained tissues were imaged with an Axio Lab.A1 microscope (Carl Zeiss MicroImaging), and images were acquired using an AxioCam ERc 5s colour camera and computer processed using AxioVision (Carl Zeiss MicroImaging).

#### Histological damage score.

Histological damage scoring was determined using criteria outlined by Wu *et al.* (2008). In brief, H&E tissue sections prepared as described above were assessed for the following damage and graded accordingly: severity of epithelial injury (0–3, from absent, to mild–superficial epithelial injury, and severe including multifocal erosions); the extent of inflammatory infiltrate (0–3, from absent to transmural); and goblet cell depletion (0–2, from absent to partial, complete). Five non-overlapping fields of view from one representative tissue section were graded from all of the six mice per treatment group and averaged to obtain a mean histological score.

#### Indirect immunofluorescence assay (IFA) on mouse colon sections.

Indirect immunofluorescence was performed on formalin-fixed paraffin-embedded (FFPE) sections and a rabbit polyclonal anti-*Citrobacter* antibody (gift from Simon Claire, Wellcome Trust Sanger Institute) was used to visualize *C. rodentium*. DNA from bacterial and intestinal epithelial cells was counterstained with Hoechst 33342. Sections were examined using an Axio Imager M1 microscope (Carl Zeiss MicroImaging), and images were acquired using an AxioCam MRm monochrome camera and computer processed using AxioVision (Carl Zeiss MicroImaging).

#### Electron microscopy.

Additional murine colonic tissues infected as described above were processed for electron microscopy, as previously described (Girard *et al.*, 2007). Samples for scanning electron microscopy (SEM) were examined without knowledge of the strain used, at an accelerating voltage of 25 kV using a JEOL JSM-5300 scanning electron microscope [JEOL (UK)]. Samples for transmission electron microscopy (TEM) were observed using a Tecnai 12 transmission electron microscope at an accelerating voltage of 120 kV (FEI). Digital pictures were taken using emmenu V3.0 (TVIPS GmbH).

### *In vivo* optical imaging of *C. rodentium*-infected mice

#### 

##### 2D bioluminescent imaging.

Whole-animal BLI was measured on days 4 and 8 p.i. using an IVIS Spectrum optical imaging system (Caliper). Mice were anaesthetized with isofluorane and each animal’s abdominal region was depilated using depilating cream prior to BLI. Regions of interest were identified and quantified (photons s^−1^ cm^−2^ sr^−1^) (sr  =  Steradian) using Living Image 4.2 software (Caliper).

##### 3D bioluminescent imaging.

A representative mouse from each group was selected for 3D imaging based upon the 2D BLI data. Mice were anaesthetized using an XGI-8 Anesthesia System (Caliper) and subsequently transferred to a mouse imaging shuttle and humanly restrained using translucent tape. The mouse imaging shuttle was placed into an IVIS Spectrum and imaged using DLIT, with emission wavelengths ranging from 520 to 560 nm with photon binning of 8 and BL image acquisition times set to automatic for each filter set to maximize the signal to noise ratio. The 3D BL optical image was then reconstructed using Living Image 4.2, utilizing the multi-modality imaging tool, as described earlier (Kuo *et al.*, 2007).

##### µCT imaging and Dicom file generation.

Following DLIT, the mouse imaging shuttle was transferred to the Quantum FX µCT imager (Caliper). The mouse imaging shuttle was positioned so that the fiducial of the imaging shuttle lined up with the fiducial in the Quantum FX µCT. A whole-mouse µCT scan was performed in two stages using a 76 mm field of view (FOV) with a voxel size of 128 µm. The µCT scan was then automatically reconstructed into a Dicom file using RigakuSW (Caliper).

##### Co-registration of DLIT-µCT data and generation of a 3D reconstruction.

Co-registration of the DLIT 3D BL images and the Dicom file containing the whole-mouse µCT scan was performed using Living Image 4.2 (Caliper). The fiducial on the mouse imaging shuttle was used to align the Dicom file with the DLIT 3D BL image to generate the final 3D DLIT-µCT reconstruction.

##### Generation of a temporal video of *C. rodentium* infection.

The representative mice from each group, described above, were imaged daily using DLIT-µCT. The subsequent 3D DLIT-µCT reconstructions were ‘stitched’ together and suitably annotated using Windows Live Movie Maker (http://windows.microsoft.com/en-GB/windows7/products/features/movie-maker) to generate a four-dimensional (4D) movie of the *C. rodentium* infection. The movies generated using this software were converted into .avi files using a file converter.

#### Statistical analysis.

All results were presented as scatter plots with the mean values. A one-way analysis of variance (ANOVA) was performed with a Tukey’s multiple comparison post-test using commercially available software (GraphPad 5, GraphPad software); a *P* value of <0.05 was taken to be significant.

## Results

### Treatment of mice with *B. breve* UCC2003 does not reduce *C. rodentium* colonization

**Fig. 1.**
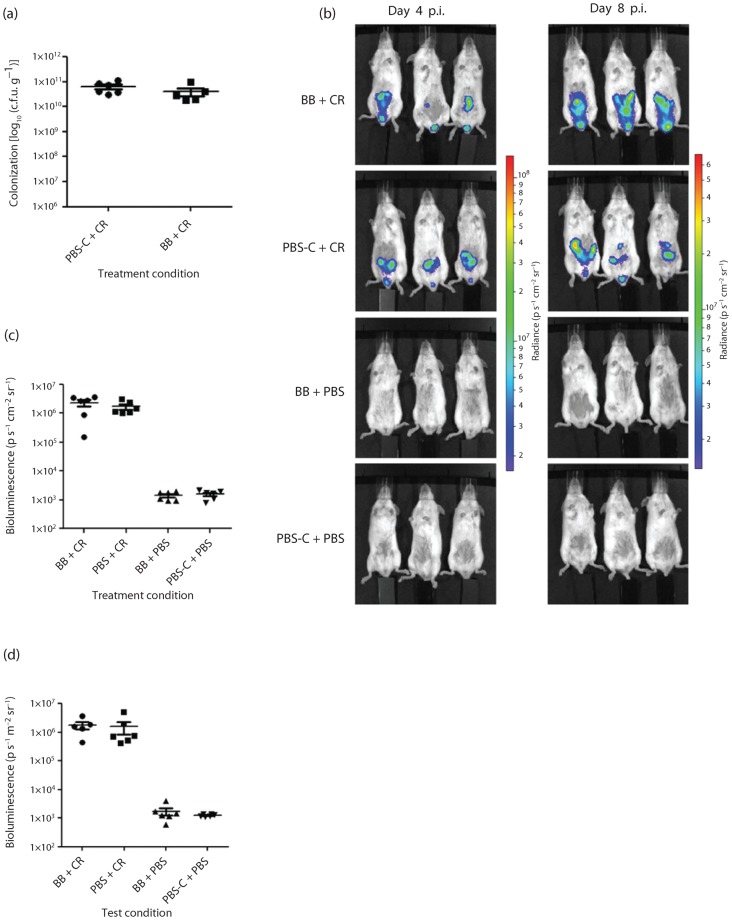
*C. rodentium* colonization dynamics following treatment of mice with *B. breve.* Mice were pre-treated for 3 days prior to *C. rodentium* infection and daily following infection with *B. breve* (BB+CR) or PBS-C (PBS-C+CR). Control mice were treated with *B. breve* and PBS mock-infected (BB+PBS), or PBS-C mock-treated and PBS mock-infected (PBS-C+PBS) as controls. (a) Quantification of *C. rodentium* c.f.u. from stools taken at day 7 p.i. (b) *In vivo* optical imaging of a bioluminescent *C. rodentium* infection from three representative mice per test condition at days 4 and 8 p.i. (c) Quantification of BLI (p s^−1^ cm^−2^ sr^−1^; p = photons) from all six mice per treatment condition at day 4 p.i. (d) Quantification of BLI (p s^−1^ cm^−2^ sr^−1^) from all six mice per test condition at day 8 p.i.

To determine whether *B. breve* could competitively exclude *C. rodentium*, mice were pre-treated for 3 days, challenged with *C. rodentium* and administered *B. breve* daily for the duration of the infection (BB+CR). As controls, mice were also challenged with *C. rodentium* and administered PBS-C daily for the duration of the infection (PBS-C+CR), mock-infected with PBS and administered *B. breve* daily (BB+PBS), or mock-infected with PBS and mock-treated with PBS-C (PBS-C+PBS). *B. breve* treatment did not significantly reduce *C. rodentium* colonization when evaluated by bacterial enumeration (Fig. 1a) or BLI (Fig. 1b–d) when compared with the PBS-C+PBS-treated group. BLI of BB+CR mice did not significantly reduce (*P*>0.05) the total BL signal (Fig. 1c, d) when compared with the PBS-C+CR-treated control. Furthermore, in line with previous reports (Wiles *et al.*, 2004), the spatial distribution of *C. rodentium* within infected mice quantified using BLI was heterogeneous between treatment groups (Fig. 1b–d), and irrespective of the treatment group demonstrated heavy *C. rodentium* colonization of the caecum at day 4 p.i. and the distal colon and rectum at day 8 p.i. (Fig. 1b). Interestingly, qualitative assessment of BLI of BB+CR mice demonstrated a shift in the BL signal towards the large intestine (Fig. 1b).

**Fig. 2.**
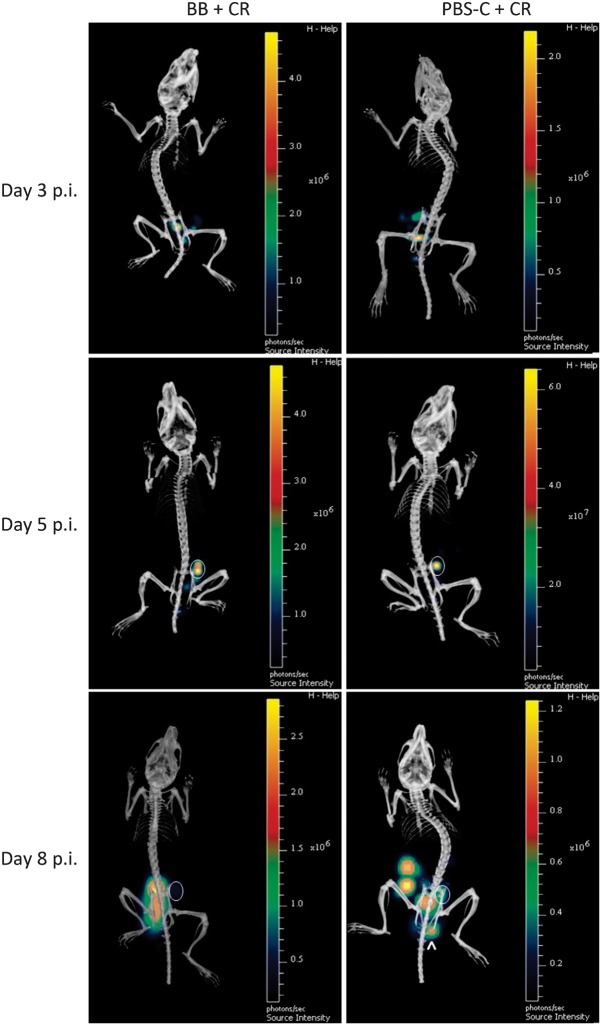
*C. rodentium* colonization dynamics following treatment of mice with *B. breve*. Mice were pre-treated for 3 days prior to *C. rodentium* infection and daily following infection with *B. breve* (BB+CR) or PBS-C (PBS-C+CR). (a) DLIT-µCT scan of bioluminescent *C. rodentium* infection from one representative mouse per group monitored at days 3, 5 and 8 p.i. Circles indicate caecal colonization; the arrowhead indicates rectal colonization.

In addition to BLI, a representative mouse from each group was selected for DLIT-µCT and *C. rodentium* colonization was monitored daily up to day 8 p.i. The DLIT-µCT data were compiled to generate a 4D movie of the BB+CR or PBS-C+CR mice. Notably, *C. rodentium* appears to be randomly distributed within the small intestine between days 1 and 4 p.i. until day 5 p.i., where the BL foci concentrate in the caecum (Fig. 2, Videos S1 and S2 available with the online version of this paper), as described previously (Wiles *et al.*, 2004). BB+CR or PBS-C+CR mice demonstrated similar DLIT-µCT profiles between days 3 and 5 p.i., and *B. breve* treatment did not affect this distinct caecal tropism observed at day 5 p.i. (Fig. 2, Videos S1 and S2). Strikingly, at day 6 p.i. in both the BB+CR and PBS-C+CR mice, *C. rodentium* appears to undergo a ‘virulence switch’ and subsequently colonizes the large intestine, which peaks at day 8 p.i. (Fig. 2, Videos S1 and S2). However, at day 8 p.i. in BB+CR mice, colonization was concentrated to the colon with a weak BL focus in the caecum, whereas in PBS-C+CR mice multiple bioluminescent foci were observed in the caecum, colon and rectum (Figs 1b and 2).

**Fig. 3.**
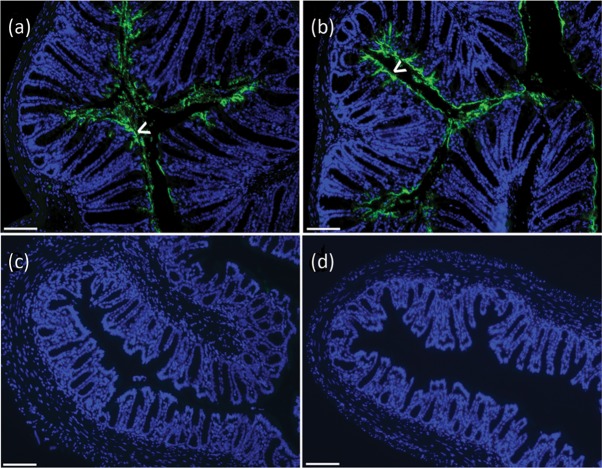
Indirect IFA of murine terminal colon taken at necropsy from mice treated with *B. breve* or PBS-C and subsequently infected with *C. rodentium* for 8 days. Mice were pre-treated for 3 days prior to *C. rodentium* infection and daily following infection with (a) *B. breve* (BB+CR) or (b) PBS-C (PBS-C+CR). Control mice were treated with (c) *B. breve* and PBS mock-infected (BB+PBS) or (d) PBS-C mock-treated and PBS mock-infected (PBS-C+PBS). *C. rodentium* colonizes the epithelial layer lining the lumen of the colon (arrowheads) irrespective of the probiotic treatment. Bars, 100 µm.

To determine whether *B. breve* treatment altered *C. rodentium* distribution within the colonic mucosa, FFPE sections of terminal colon taken during necropsy at the peak of bacterial infection (day 8 p.i.) were investigated by an indirect IFA. In line with previous reports (Crepin *et al.*, 2010), *C. rodentium* colonized epithelial cells lining the colonic lumen and bacteria did not penetrate into the colonic crypts (Fig. 3b). BB+CR and PBS-C+CR mice demonstrated identical colonization by IFA (Fig. 3a, b).

### Treatment of mice with *B. breve* does not reduce A/E lesion formation

**Fig. 4.**
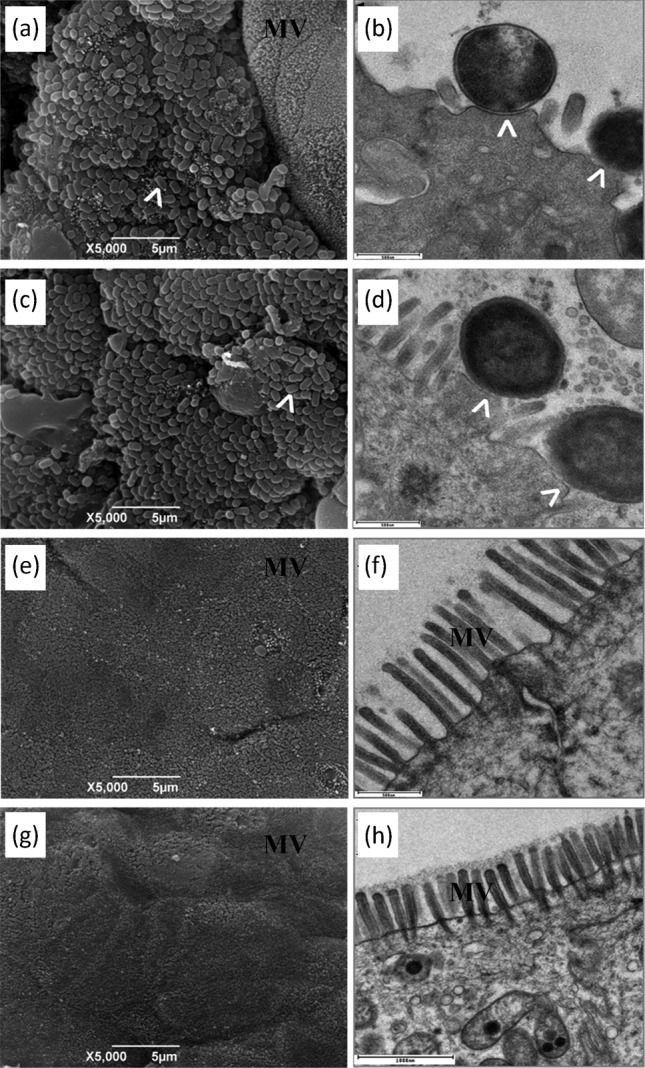
SEM (a, c, e, g) and TEM (b, d, f, h) of murine terminal colon taken at necropsy from mice treated with *B. breve* or PBS and subsequently infected with *C. rodentium* for 8 days. Mice were pre-treated for 3 days prior to *C. rodentium* infection and daily following infection with *B. breve* (a, b), PBS (c, d), or were *B. breve*-treated and PBS mock-infected (e, f) or PBS-C mock-treated and PBS mock-infected as a negative control (g, h). *C. rodentium* infection induces A/E lesion (arrowheads) formation irrespective of the probiotic treatment. MV, microvilli.

We determined whether the mechanisms utilized by *C. rodentium* to colonize the intestinal mucosa had been altered by *B. breve* treatment. A/E lesion formation on sections of terminal colon taken at necropsy at the peak of bacterial infection (day 8 p.i.) were investigated qualitatively by SEM and TEM. Prophylactic treatment of mice with *B. breve* did not affect A/E lesion formation (Fig. 4a, e) when compared with the BB+PBS- and PBS-C+PBS-treated controls. Examination of the colonic mucosa of BB+PBS mice for A/E lesions by SEM demonstrated no adverse pathology (Fig. 4c, g) and resembled the tissues from PBS-C+PBS-treated mice.

### Prophylactic treatment of mice with *B. breve* significantly reduces *C. rodentium*-associated pathology

**Fig. 5.**
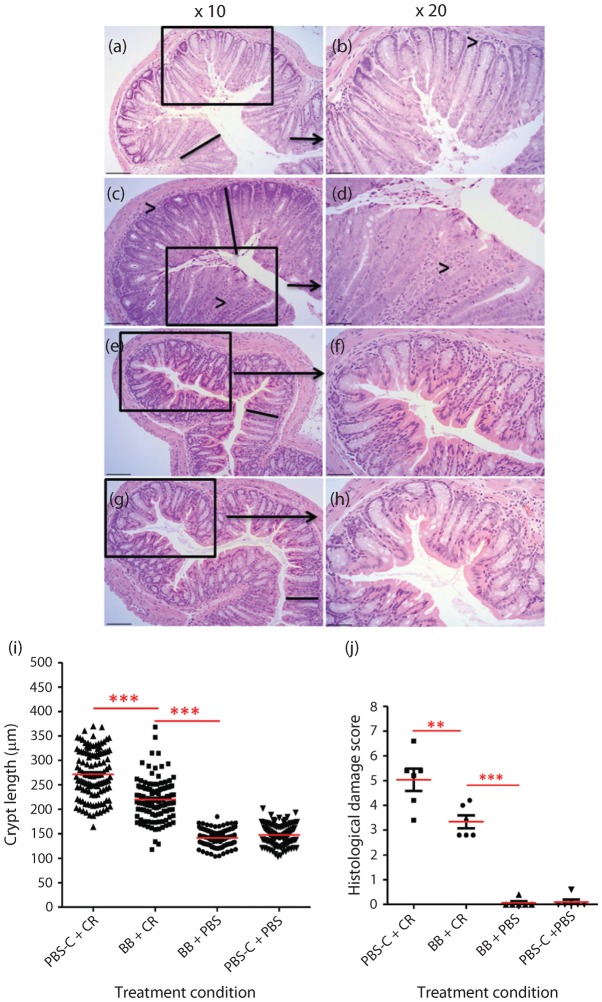
Evaluation of CCH (black lines) in mice treated with *B. breve* and subsequently infected with *C. rodentium* for 8 days. Mice were pre-treated for 3 days prior to *C. rodentium* infection and daily following infection with *B. breve* (BB+CR; a, b) or PBS-C (PBS-C+CR; c, d). Control mice were treated with *B. breve* and PBS mock-infected (BB+PBS; e, f) or PBS-C mock-treated and PBS mock-infected (PBS-C+PBS; g, h). *B. breve* treatment significantly reduced *C. rodentium*-induced pathology and lymphocyte accumulation in the lamina propria (arrowheads). Bars: ×10, 100 µm; ×20, 65 µm. (i) Quantification of crypt hyperplasia following treatment of mice with *B. breve* or PBS. Treatment of mice with *B. breve* significantly (*P*<0.001, ***) reduced crypt hyperplasia when compared with PBS-treated mice. (j) Histological damage score of H&E-stained colonic sections demonstrating a significant reduction (*P*<0.01, **; *P*<0.001, ***) in *C. rodentium*-associated pathology following treatment with *B. breve*.

Although probiotic treatment was unable to competitively exclude *C. rodentium*, other parameters of an infection such as tissue pathology play an important role in the disease severity and clinical symptoms. Histopathological analyses of sections of terminal colon taken at necropsy at the peak of bacterial infection (day 8 p.i.) were investigated by examination of H&E-stained tissues by light microscopy (Fig. 5a–j). Quantification of colonic crypt hyperplasia (CCH) demonstrated that *B. breve* significantly reduced colonic crypt length (*P*<0.001) when compared with the PBS-C+CR control group, and the difference between the arithmetic means of BB+CR and PBS-C+CR mice was 219.82±40.71 µm and 271.49±48.82 µm, respectively (Fig. 5i). *B. breve* treatment did not completely inhibit CCH, and BB+CR mice demonstrated significantly (*P*<0.001) more CCH than BB+PBS controls (Fig. 5i). Qualitative assessment of H&E-stained tissues using a histological damage score demonstrated a significant reduction (*P*<0.01) in *C. rodentium*-associated pathology in BB+CR mice when compared with the PBS-C+CR control group (Fig. 5j). Importantly, there was a qualitative reduction in immune cell infiltration of the lamina propia in BB+CR mice when compared with PBS-C+CR mice (Fig. 5a–d), suggesting antiinflammatory activity of *B. breve*.

## Discussion

Probiotics, in particular members of the genera *Lactobacillus* and *Bifidobacterium*, are widely used to treat gastrointestinal disease caused by enteric pathogens, especially in the developing world (Huebner & Surawicz, 2006; Picard *et al.*, 2005; Preidis *et al.*, 2011). The *C. rodentium* infection model is now routinely used to test the efficacy of putative probiotic strains and the mechanisms by which such strains confer protective effects upon the host (Chen *et al.*, 2005, 2009; D’Arienzo *et al.*, 2006; Fanning *et al.*, 2012; Gareau *et al.*, 2010; Johnson-Henry *et al.*, 2005; Rodrigues *et al.*, 2012; Wu *et al.*, 2008). Our results demonstrate that the prophylactic administration of *B. breve* UCC2003 to mice did not prevent *C. rodentium* colonization or prevent A/E lesion formation, but significantly (*P*<0.001) reduced CCH and *C. rodentium*-associated pathology, including the infiltration of inflammatory cells into the colonic mucosa. Recently, a study by Jones & Knight (2012) demonstrated that *Bacillus subtillus* could reduce CCH caused by *C. rodentium* infection and that this immunomodulatory effect was independent of a reduction in pathogen colonization. In contrast, several groups have reported that the administration to mice of a variety of probiotic micro-organisms including *Lactobacillus acidophilus*, *B. breve* and *S. boulardii* in PBS could competitively exclude *C. rodentium*, largely through unknown mechanisms (Chen *et al.*, 2005, 2009; Fanning *et al.*, 2012; Wu *et al.*, 2008). The mechanistic differences observed in these studies are likely due to a combination of the different probiotic micro-organisms, mouse strains and age of the animals used. Importantly, Fanning *et al.* (2012) demonstrated that *B. breve* could competitively exclude *C. rodentium* directly through the production of bacterial exopolysaccharide, presumably by competing with *C. rodentium* for host cell receptors on the caecal and colonic epithelium. That study used 6–8-week-old BALB/c mice, which demonstrated altered *C. rodentium* colonization dynamics that rapidly peaked by day 4 p.i. (Fanning *et al.*, 2012), unlike the more widely used C57 BL/6 model, which peaks at day 8 p.i. (Rodrigues *et al.*, 2012). Moreover, the authors of that study did not report data concerning CCH; however, future studies should focus on why we observe such dramatic differences in the CE effects of *B. breve* for *C. rodentium* between these two different mouse strains and how this impacts on the colonic inflammatory response.

In this study we generated the first 4D infection movie using DLIT-µCT and successfully used this technology to visualize how *B. breve* affects *C. rodentium* colonization up to the peak of bacterial infection (day 8 p.i.). The 4D movies facilitated the analysis of a large volume of data in a quick and easy to interpret format, and provided a unique insight into the development of *C. rodentium* infection. In particular, we observed a novel ‘virulence switch’ between days 5 and 6 p.i., whereby *C. rodentium* forms a focus of infection in the caecum at day 5, which then expands as the bacteria colonize the large intestine at day 6 p.i. It is tempting to speculate that *C. rodentium* is adapting to the caecal microenvironment, possibly by altering its metabolism, which allows the bacteria to multiply rapidly and colonize the large intestine.

Collectively, these data demonstrate that *B. breve* can modulate the murine colonic inflammatory response through unknown mechanisms. Further work is required to determine the underlying molecular mechanisms behind the reduction in *C. rodentium*-induced CCH and lymphocyte infiltration caused by the prophylactic administration of *B. breve*.
